# Multimodal deep learning to support delineation of neuro-oncology organs based on the EPTN international neurological contouring atlas

**DOI:** 10.1016/j.phro.2026.101051

**Published:** 2026-07-29

**Authors:** Ana M. Barragán-Montero, Margerie Huet-Dastarac, Dario Di Perri, David Hofstede, Nikolina E. Birimac, Matijs Geerts, Benjamin Roberfroid, Emilien Quéré, John A. Lee, Erik Roelofs, Wouter van Elmpt, Daniëlle B.P. Eekers, Catharina M.L. Zegers

**Affiliations:** a UCLouvain – Institut de Recherche Expérimentale et Clinique - Molecular Imaging Radiotherapy and Oncology (MIRO), Brussels, Belgium; b Department of Radiation Oncology, Cliniques Universitaires Saint-Luc, Brussels, Belgium; c École nationale supérieure de techniques avancées Bretagne (ENSTA), Brest, France; d Department of Radiation Oncology (Maastro), GROW Research Institute for Oncology and Reproduction, Maastricht University Medical Centre+, Maastricht, the Netherlands

**Keywords:** Artificial intelligence, autosegmentation, EPTN atlas, neuro-oncology, nnU-Net

## Abstract

We present a multimodal deep learning model for segmenting 25 organs defined in the European Particle Therapy Network (EPTN) international neurological contouring atlas. Multiple input configurations were evaluated on 74 patients using 5-fold cross-validation (59 training, 14–15 per fold for evaluation), with each patient assessed once on unseen data. The dual-input model combining contrast-enhanced T1-weighted magnetic resonance (MR) and computed tomography (CT) achieved the best overall results (median Dice of 0.80, median surface Dice of 0.84), with no added benefit from T2 FLAIR.

## Highlights

• Multimodal deep learning model segmented 25 neuro-oncology organs.
• The best model combined T1-weighted magnetic resonance and computed tomography.
• Median surface Dice of 0.84 demonstrated robust performance across diverse organs.
• The model supports segmentation assistance and retrospective dose-response analyses.

## Graphical abstract

**Figure:**
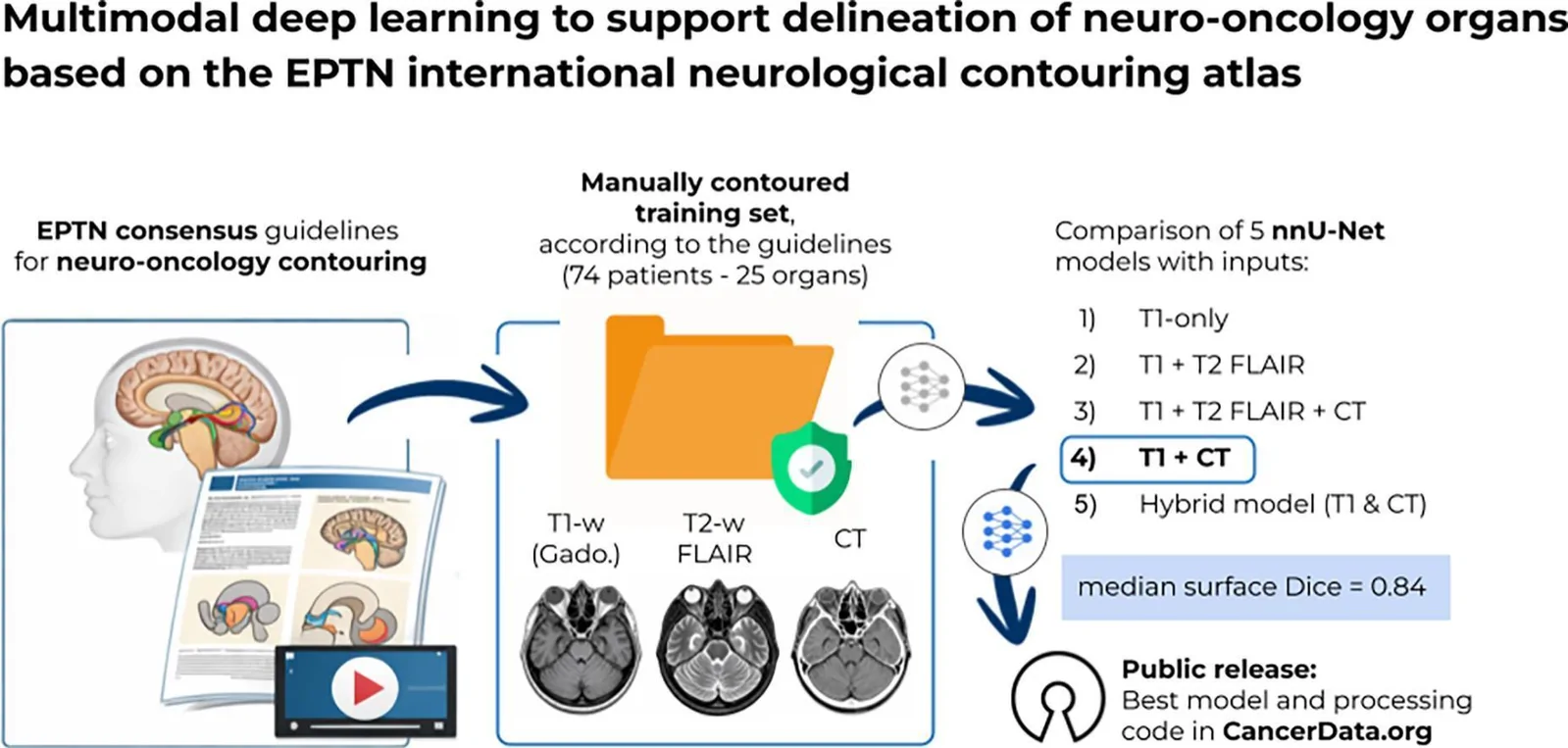

## 1 Introduction

Accurate delineation of organs-of-interest (OOIs) is critical in radiotherapy treatment planning, particularly where radiation-induced side effects can have dramatic consequences for the patient. Beyond accuracy, consistent OOI delineation is essential to: 1) standardize clinical practice and ensure equitable treatment quality across different clinicians and institutions; and 2) enable reliable side effects data pooling in multicenter clinical trials to support evidence-based dose guidance.

Neuro-oncology radiotherapy involves a large number of OOIs, including structures associated with cognitive function and late side effects [1], [2], [3], [4], [5]. Significant efforts have been made to harmonize OOIs contouring through consensus-based guidelines and accompanying educational resources, such as the European Particle Therapy Network (EPTN) neurological contouring atlas [6], [7], [8], [9], [10], [11], [12], [13]. Despite this, many institutions continue to omit some of these OOIs, primarily due to time constraints and their inherent complexity.

In this context, artificial intelligence (AI) offers a promising solution to facilitate broad implementation of standardized contouring atlases. When trained on high-quality data [14], [15], [16], AI models can encode complex anatomical knowledge and replicate expert-level contouring with high reproducibility. This work investigates the performance of a deep learning (DL) model trained on reference contours for 25 OOIs delineated as defined in the EPTN neurological contouring atlas [6], [7], [8], [9], [10], [11], with a focus on identifying the optimal input image modality configuration. We evaluated whether a model trained on T1-weighted magnetic resonance (MR) and computed tomography (CT) achieved superior performance for prospective clinical validation and use. The model and dedicated scripts are made publicly available, serving as a complementary tool alongside traditional text and video-based resources to support the dissemination of contouring guidelines. Although originally developed in the context of particle therapy, the EPTN atlas and the present model are broadly applicable to neuro-oncology radiotherapy regardless of treatment modality.

## 2 Materials and methods

### 2.1 Database

The dataset included 74 patients with primary intracranial tumours (mainly gliomas and meningiomas, Supplementary Table S1), treated at the Maastro Department of Radiotherapy (Maastricht, The Netherlands) between 2019 and 2021. Ethical approval was obtained from the Internal Review Board (project number: W 23 0200069).

The cohort had a median age of 50.5 years (range [19, 91] years), with a sex distribution of 51% female and 49% male. It exhibited anatomical variations including surgical resections (Supplementary Fig. S1), or enlarged volumes associated with hydrocephalus. For each patient, available data included a planning CT with intravenous iodine contrast, 3 Tesla MR (gadolinium-enhanced T1-weighted and T2-weighted FLAIR), and delineations of 25 OOIs from the EPTN atlas, here referred to as reference contours. CT and MR images were rigidly registered. Reference contours were performed by two medical students trained in neuro-oncological OOI delineation based on the atlas, with selective review and correction (i.e. challenging or uncertain cases) by one clinical expert involved in the atlas development.

### 2.2 Deep learning models

We used the 3D full-resolution configuration of nnU-Net [17], state-of-the-art for biomedical image segmentation [18]. Five different settings were explored, using different input channel combinations: 1) T1-only; 2) T1 + T2 FLAIR; 3) T1 + T2 FLAIR + CT; 4) T1 + CT; and 5) Hybrid model (T1 & CT) combining predictions from two independently trained models: one using T1 only and the other using CT only**.**

While models in settings 1 to 4 segmented all organs together from the given input images, the fifth setting was designed taking into account that certain OOIs are better visualized on CT, while others benefit from MR. Structures segmented by the CT-based model in setting 5 were: brain, vestibule and semicircular canals (VSCCs), corneas, cochleas, lacrimal glands, lenses, retinas, maculas, and foveas. The remaining structures were segmented using the T1-based model.

### 2.3 Data preprocessing

As nnU-Net assigns a unique integer value to each voxel, non-overlapping reference contours were required for training. Thus, a structure-priority scheme was defined by the clinicians to minimize information loss in regions where contours might overlap, reflecting each structure's clinical importance based on associated side effects [19]. The following order (increasing priority) was used for overlap removal: brain, ventricles, cerebellum, corpus callosum, orbitofrontal cortex, caudate nuclei, amygdalae, VSCCs, corneas, cochleas, thalami, fornices, hypothalami, hippocampi, lacrimal glands, lenses, retinas, maculas, foveas, pituitary gland, pineal gland, optic tracts, optic nerves, brainstem, and optic chiasm. Bilateral (left/right) and multi-part (anterior/posterior) structures defined separately in the atlas were merged to reduce model complexity, yielding the above-listed 25 structures.

For each patient, MRIs were resampled to match the CT voxel spacing and image size (image acquisition details in Supplementary Material A). All subsequent pre-processing (e.g. normalization, resampling) and data augmentation, were performed automatically by nnU-Net's standard pipeline (version v2.2) [20].

### 2.4 Model training, selection, and evaluation

Models were trained on a NVIDIA TeslaA100 GPU (80 GB RAM), using 5-fold cross-validation with 80% of data (59 patients) for training and 20% (15 patients in one fold, 14 in the others) held-out for evaluation. No separate test set was used due to the limited dataset size. Therefore, for each fold, the model at the last training epoch (epoch 1000, nnU-Net's default) was selected, without early stopping or epoch selection based on held-out performance. Performance was then evaluated using the held-out data of each cross-validation split, such that each patient was assessed only once on unseen data, resulting in 74 independent predictions.

Dice similarity coefficient (DSC), surface DSC (sDSC) [21], added path length (APL) [22], and Hausdorff Distance at percentile 95% (HD95) were used to quantify model performance (library details in Supplementary Material B). sDSC was computed using a tolerance threshold of 1 voxel for all structures.

Statistical comparisons were performed independently for each organ using paired Wilcoxon signed-rank tests; no correction for multiple testing was applied, as analyses aimed to identify performance patterns across organs, rather than to test a single predefined hypothesis.

### 2.5 Public code and model

Python code for data processing (nifti format conversion and image registration based on OpenTPS [27], [28] libraries) and model inference, as well as the best-performing model (T1 + CT), and the corresponding AID-RT (Artificial Intelligence Documentation in RadioTherapy) model card [23], [24], [25] have been published at CancerData [26].

## 3 Results

**Fig. 1:**
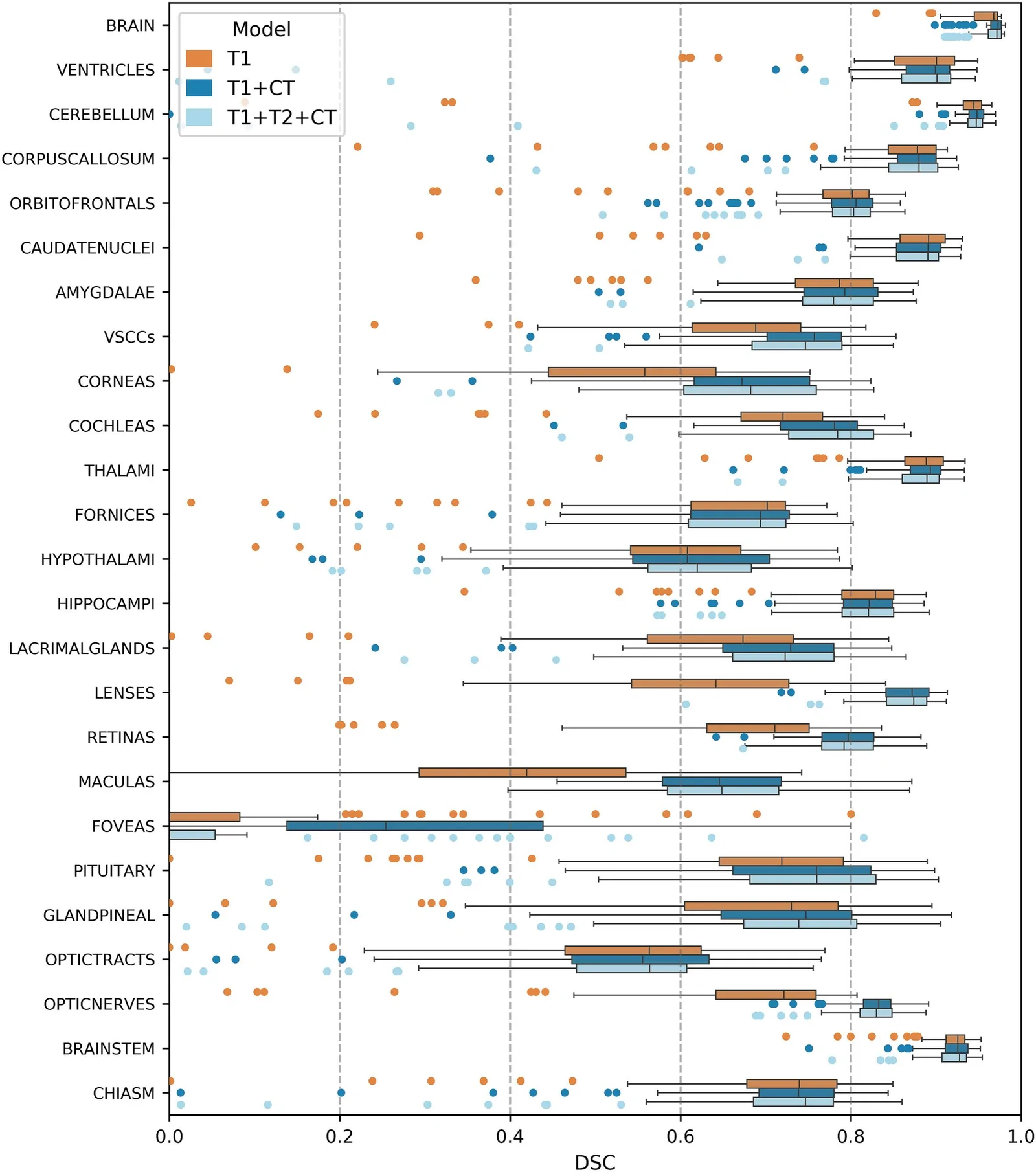
Boxplots of the Dice Similarity Coefficient (DSC) per organ for the T1, T1 + T2 + CT, and T1 + CT models, computed using reference contours after overlap removal across 74 patients. The median is indicated by a vertical line within the boxes, boxes represent the interquartile range, and whiskers extend to 1.5 × the interquartile range; outliers are shown as individual points.

The T1 + CT model achieved the best overall performance, with median DSC, sDSC, APL and HD95 of 0.80, 0.84, 647 and 2.24 mm, respectively; followed by the T1 + T2 + CT and Hybrid model (Table 1, Fig. 1). Per-organ metric values, computed using reference contours after overlap removal according to the defined structure-priority scheme, are reported in Supplementary Tables S2–5. The T1 + CT model showed robustness to complex anatomies, such as surgical resections (Supplementary Fig. S1).

**Table 1:** Median and interquartile range (IQR) of Dice similarity coefficient (DSC), surface DSC (sDSC), added path length (APL), and Hausdorff Distance at percentile 95% (HD95), computed across all 25 organs and 74 patients for each model. All metrics were computed using reference contours after overlap removal. Bold numbers indicate the best achieved value per metric. Arrows indicate the direction of improvement (↑ higher is better, ↓ lower is better).

	**T1**	**T1+T2**	**T1+T2+CT**	**T1+CT**	**Hybrid (T1** **+** **CT)**
**DSC↑**	0.74 [0.60–0.86]	0.74 [0.60–0.85]	**0.80** **[0.69–0.87]**	**0.80** **[0.69–0.87]**	0.78 [0.65–0.87]
**sDSC↑**	0.78 [0.64–0.87]	0.78 [0.63–0.87]	0.83 [0.73–0.91]	**0.84** **[0.74–0.91]**	0.82 [0.68–0.90]
**APL (mm)↓**	748 [272–3684]	740 [274–3668]	657 [240–3413]	**647** **[239–3409]**	709 [234–3453]
**HD95 (mm)↓**	2.83 [2.0–4.12]	2.83 [2.0–4.12]	2.24 [1.73–3.61]	**2.24** **[1.73–3.44]**	2.24 [1.73–3.61]

The T1 + CT model was statistically significantly superior to the Hybrid model in 7 organs (*p* < 0.001 for optic nerves, pituitary, foveas, maculas, cochleas, VSCCs, and cerebellum); including striking differences in CT-visible structures: cochleas (median DSC 0.78 vs 0.69), maculas (0.65 vs 0.00), and foveas (0.25 vs 0.00) (Supplementary Fig. S2). Adding CT led to a clear improvement over T1 and T1 + T2 models, with an increase in median DSC larger than 0.1 for corneas, lenses, maculas, and foveas (Supplementary Table S2). T2 FLAIR provided negligible benefit over T1 alone, with differences in median DSC below 0.015.

Median sDSC scores were higher than DSC for small organs (>0.1 in multiple organs) and lower for larger ones (e.g., brain, cerebellum). For most organs, overlap removal affected less than 1% of volume (median) of the reference contours, with higher removal for a few structures (up to ∼17% for the brain; Table S7). The resulting impact on DSC/sDSC remained below 0.03 for all organs except the brain, which is processed first and thus contains holes from sequential organ removal (Supplementary Fig. S3, Table S6–8).

## 4 Discussion

This work presented a DL model for automated delineation of 25 organs defined in the EPTN atlas, potentially serving as a support tool for disseminating contouring guidelines. This model could assist segmentation under appropriate human supervision, and fully automated retrospective dose–response analyses (e.g., normal-tissue complication probabilities, NTCP) where manual contouring is not feasible, facilitating the development of evidence-based dose constraints.

The T1 + CT model showed the best overall performance, while adding T2 FLAIR did not provide further benefit. The paradoxical underperformance of the Hybrid model for CT-visible structures (cochleas, maculas, foveas) was attributed to training instability caused by extreme class imbalance when training exclusively on small structures (Supplementary Material C).

Limitations of this study included the absence of pediatric patients, which may limit the model's generalisability to this population; and the use of single-institution data for training and evaluation. In addition, performance was assessed using cross-validation rather than an independent external test set, which may be appropriate for ranking the considered input-channel configurations but may lead to optimistic performance estimates. External validation on independent data remains important to confirm model generalisability. Furthermore, qualitative assessment of contour quality and its influence on dose distribution, as well as proper commissioning, are necessary before clinical deployment.

Another limitation of this study was the absence of explicit interobserver variability (IOV) analysis, which would provide a clinically meaningful benchmark for interpreting model performance and its potential for standardizing contouring practices. Available literature shows evidence of structure-dependent IOV. Lorenzen et al. [29] reported DSC values from 0.71 for small structures like optic chiasm to 0.89 for larger ones like the brainstem. Chamunyonga et al. [30] observed lower agreement, with DSC as low as 0.39 for the optic chiasm among students and qualified radiation therapists. Similarly, Vogin et al. [31] reported substantial variability for small structures such as the optic chiasm and cochlea. This suggests that the lower performance observed for some small structures in our study may partly reflect intrinsic IOV rather than solely model limitations.

While DSC remains the most commonly used metric in literature, it has known limitations for small structures and it is recommended to complement it with boundary-based metrics [32], [33]. sDSC and APL might be therefore preferred here, as they provide this boundary-based complement and have shown to correlate better with contouring time savings, making them more clinically meaningful [22], [34].

Note that some structures are neither visible on MR nor CT images: the macula is defined relative to the retina and optic nerves, and the periventricular space (PVS) through geometric expansion of the ventricles. PVS was not addressed here as it was investigated separately [35] and could be derived from the present model's ventricle segmentation. For the macula, rule-based post-processing leveraging retina and optic nerves segmentation could improve current results. Furthermore, standardizing eye closure instructions during imaging could reduce optical structure motion [36], potentially improving annotation consistency and model performance.

Previous work about auto-segmentation of neuro-oncology organs included commercial or in-house atlas-based and DL models [35], [37], [38], [39], [40], [41], [42], [43]. Performance was consistently high for large structures (brainstem DSC ∼0.89–0.96, cerebellum ∼0.92–0.95) [37], [38], [42], and lower, more variable for smaller ones (optic chiasm 0.53–0.83, hippocampus 0.66–0.93) [37], [39], [40], [41]. Some models outperformed ours for specific structures, notably Crouzen et al. [40] for hypothalami (0.78 vs 0.61) and optic chiasm (0.83 vs 0.74), and Mekki et al. [38] for hippocampi (0.93 vs 0.82), though direct comparisons are limited by differences in imaging protocols and reference contours quality. In contrast, our T1 + CT model outperformed Vaassen et al. [41] for optic chiasm (0.74 vs 0.53) and hippocampus (0.82 vs 0.72), and Turcas et al. [37] across most structures, including hippocampi (0.82 vs 0.68), optic chiasm (0.74 vs 0.55), and optic nerves (0.83 vs 0.52). Our model also achieved comparable median DSC to Poel et al. [39] (0.80 vs 0.78). On the other hand, SynthSeg [43], widely used for neuroimaging brain parcellation rather than radiotherapy delineation, reported only global mean DSC (0.84–0.91), precluding direct per-structure comparison. Our work extends beyond prior studies by specifically covering structures in the EPTN atlas.

While the published model can be directly used for research, clinical deployment is possible but requires compliance with applicable medical device and AI regulations. Each clinical team is responsible for meeting relevant requirements, which in the EU include the Medical Device Regulation alongside AI Act. While still uncommon, there are emerging examples of publicly available in-house AI models in radiotherapy [44] and national-level implementations [45], demonstrating the feasibility of regulatory-compliant deployment of in-house models.

## Declaration of generative AI and AI-assisted technologies in the manuscript preparation

During the preparation of this work the author(s) used ChatGPT in order to correct grammar and improve writing style. After using this tool/service, the authors reviewed and edited the content as needed and take full responsibility for the content of the published article.

## CRediT authorship contribution statement

**Ana M. Barragán-Montero:** Writing – review & editing, Writing – original draft, Visualization, Validation, Software, Resources, Methodology, Investigation, Formal analysis, Data curation, Conceptualization. **Margerie Huet-Dastarac:** Visualization, Validation, Software, Resources, Methodology, Investigation, Formal analysis, Data curation, Conceptualization. **Dario Di Perri:** Writing – review & editing, Visualization, Methodology, Formal analysis, Data curation. **David Hofstede:** Writing – review & editing, Data curation. **Nikolina E. Birimac:** Writing – review & editing, Data curation. **Matijs Geerts:** Software, Data curation. **Benjamin Roberfroid:** Software. **Emilien Quéré:** Software, Formal analysis, Data curation. **John A. Lee:** Writing – review & editing. **Erik Roelofs:** Writing – review & editing, Software, Resources. **Wouter van Elmpt:** Writing – review & editing, Resources, Methodology, Conceptualization. **Daniëlle B.P. Eekers:** Writing – review & editing, Methodology, Data curation, Conceptualization. **Catharina M.L. Zegers:** Writing – review & editing, Project administration, Methodology, Formal analysis, Data curation, Conceptualization.

## Declaration of competing interest

The authors declare that they have no known competing financial interests or personal relationships that could have appeared to influence the work reported in this paper.

## Appendix A Supplementary data

Supplementary material: Patient characteristics, imaging acquisition parameters, per-organ and per-model metric tables (DSC, surface DSC, APL, HD95), overlap removal analysis, reference contour volumes, and training stability analysis of the CT Hybrid model.
