# Supplementary material for: Multimodal deep learning to support delineation of neuro-oncology organs based on the EPTN international neurological contouring atlas
Source: Phys Imaging Radiat Oncol. 2026 Jul 29;40:101051. doi: 10.1016/j.phro.2026.101051 (PMC13495586; doi:10.1016/j.phro.2026.101051)

Supplementary Material for “Multimodal deep learning to support delineation of neuro-oncology organs based on the EPTN international neurological contouring atlas”

Table S1. Patient characteristics for the patients included in the study. For each patient, tumour location, hemisphere, tumor type, surgical intervention, and treatment received are reported. Treatment abbreviations: PT = photon therapy; PH = proton therapy; PT+PH = combined photon and proton therapy.

ID	Location	Hemisphere	Type	Surgery	Treatment
1	Right	Temporal lobe	Meningioma	Yes	PH
2	Left	Cerebral meninges – parietal lobe	Meningioma	Yes	PT+PH
3	Left	Temporal lobe	Astrocytoma	Yes	PT+PH
4	Right	Vestibular schwannoma	Acoustic neuroma	Yes	PH
5	Left	Frontal lobe	Acoustic neuroma	Yes	PH
6	Right	Frontal lobe	Oligodendroglioma	Yes	PT
7	Bilateral	Pituitary region	Craniopharyngioma	Yes	PT+PH
8	Right	Cerebrum	Oligodendroglioma	Yes	PH
9	Central	Cerebellum	Ependymoma	Yes	PH
10	Left	Frontal lobe	Oligodendroglioma	Yes	PT+PH
11	Right	Cerebrum	Glioblastoma	Yes	PH
12	Left	Frontal lobe	Oligodendroglioma	Yes	PT+PH
13	Right	Frontal lobe	Astrocytoma	Yes	PT+PH
14	Left	Parietal lobe	Oligodendroglioma	Yes	PT+PH
15	Left	Temporal lobe	Astrocytoma II	Yes	PH
16	Right	Temporal lobe	Meningioma	Yes	PT+PH
17	Right	Cerebellopontine angle	Meningioma	Yes	PH
18	Right	Frontal lobe	Oligodendroglioma	Yes	PT+PH
19	Right	Pituitary region	Pituitary adenoma	Yes	PH
20	Right	Frontal lobe	Glioblastoma	Yes	PH
21	Left	Temporoparietal lobe	Astrocytoma	Yes	PT+PH
22	Left	Temporal lobe	Oligodendroglioma	Yes	PT+PH
23	Right	Frontal lobe	Oligodendroglioma	Yes	PT+PH
24	Right	Parietal lobe	Glioma	Yes	PT+PH
25	Left	Vestibular schwannoma (cerebellopontine angle)	Acoustic neuroma	No	PH
26	Right	Temporal lobe	Astrocytoma	Yes	PT+PH
27	Bilateral	Cerebellum	Meningioma	Yes	PH
28	Right	Frontotemporal lobe	Astrocytoma	Yes	PH
29	Right	Optic nerve meningioma	Meningioma	No	PT+PH
30	Left	Frontal lobe	Oligodendroglioma	Yes	PT+PH
31	Central	Frontal lobe	Astrocytoma	No	PH
32	Right	Optic nerve meningioma	Meningioma	No	PH
33	Left	Frontal lobe	Meningioma	Yes	PT+PH
34	Left	Frontal lobe	Astrocytoma	Yes	PT+PH
35	Bilateral	Pituitary region	Pituitary adenoma	Yes	PT
36	Left	Temporal lobe	Glioblastoma	Yes	PH

37	Right	Temporal lobe	Astrocytoma	Yes	PT+PH
38	Right	Cavernous sinus	Meningioma	No	PH
39	Left	Frontal lobe	Astrocytoma	Yes	PT+PH
40	Right	Frontal lobe	Low-grade glioma	No	PT
41	Right	Frontal lobe	Glioblastoma	Yes	PH
42	Bilateral	Suprafrontal lobe / pituitary region	Craniopharyngioma	No	PT+PH
43	Right	Frontal lobe	Glioblastoma	No	PH
44	Right	Frontal lobe	Oligodendroglioma	Yes	PT+PH
45	Left	Frontal lobe	Glioblastoma	Yes	PH
46	Left	Temporal lobe	Astrocytoma	Yes	PT+PH
47	Right	Frontal lobe	Meningioma	Yes	PT
48	Right	Frontal lobe	Mesenchymal chondrosarcoma	Yes	PT+PH
49	Left	Frontal lobe	Oligodendroglioma	Yes	PT+PH
50	Left	Frontal lobe	Meningioma	Yes	PT+PH
51	Left	Frontal lobe	Oligodendroglioma	Yes	PT+PH
52	Right	Frontal lobe	Oligodendroglioma	Yes	PT+PH
53	Bilateral	Frontal lobe	Oligodendroglioma	Yes	PT
54	Left	Parietal lobe	Oligodendroglioma	Yes	PT+PH
55	Right	Frontal lobe	Astrocytoma	Yes	PT+PH
56	Left	Parietal lobe	Astrocytoma	Yes	PT+PH
57	Left	Frontal lobe	Astrocytoma	Yes (biopsy only)	PT+PH
58	Left	Glomus jugulotympanicum	Paraganglioma	Yes	PH
59	Right	Skull base	Meningioma	Yes	PH
60	Left	Temporal lobe	Glioblastoma	Yes	PH
61	Right	Temporal lobe	Oligodendroglioma	Yes	PT
62	Right	Vestibular schwannoma	Acoustic neuroma	Yes	PH
63	Right	Temporal lobe	Hemangiopericytoma	Yes	PH
64	Right	Temporal lobe	Oligodendroglioma	Yes	PT+PH
65	Left	Frontal lobe	Oligodendroglioma	Yes	PT
66	Left	Pituitary region	Pituitary adenoma	Yes	PT+PH
67	Right	Vestibular schwannoma	Acoustic neuroma	Yes	PH
68	Left	Frontal lobe	Oligodendroglioma	Yes	PT+PH
69	Right	Parietal lobe	Gliosarcoma	Yes	PH
70	Right	Frontal lobe	Oligodendroglioma	Yes	PT+PH
71	Left	Parietal lobe	Meningioma	Yes	PT+PH
72	Right	Vestibular schwannoma	Acoustic neuroma	No	PH
73	Left	Clivus	Meningioma	No	PH
74	Bilateral	Occipital lobe	Hemangiopericytoma	Yes	PT+PH

Figure S1: Illustrative axial slices from three patients with surgical resections. Orange arrows highlight the resection cavities. Yellow contours represent the reference contours and blue contours are the predicted contours with the T1+CT model. A) Patient with resection involving the right amygdala and hippocampus. B) Patient with resection involving the left amygdala and part of the hippocampus. C) Patient with resection involving the anterior corpus callosum, and the right orbitofrontal region, with associated ventricular deformation.

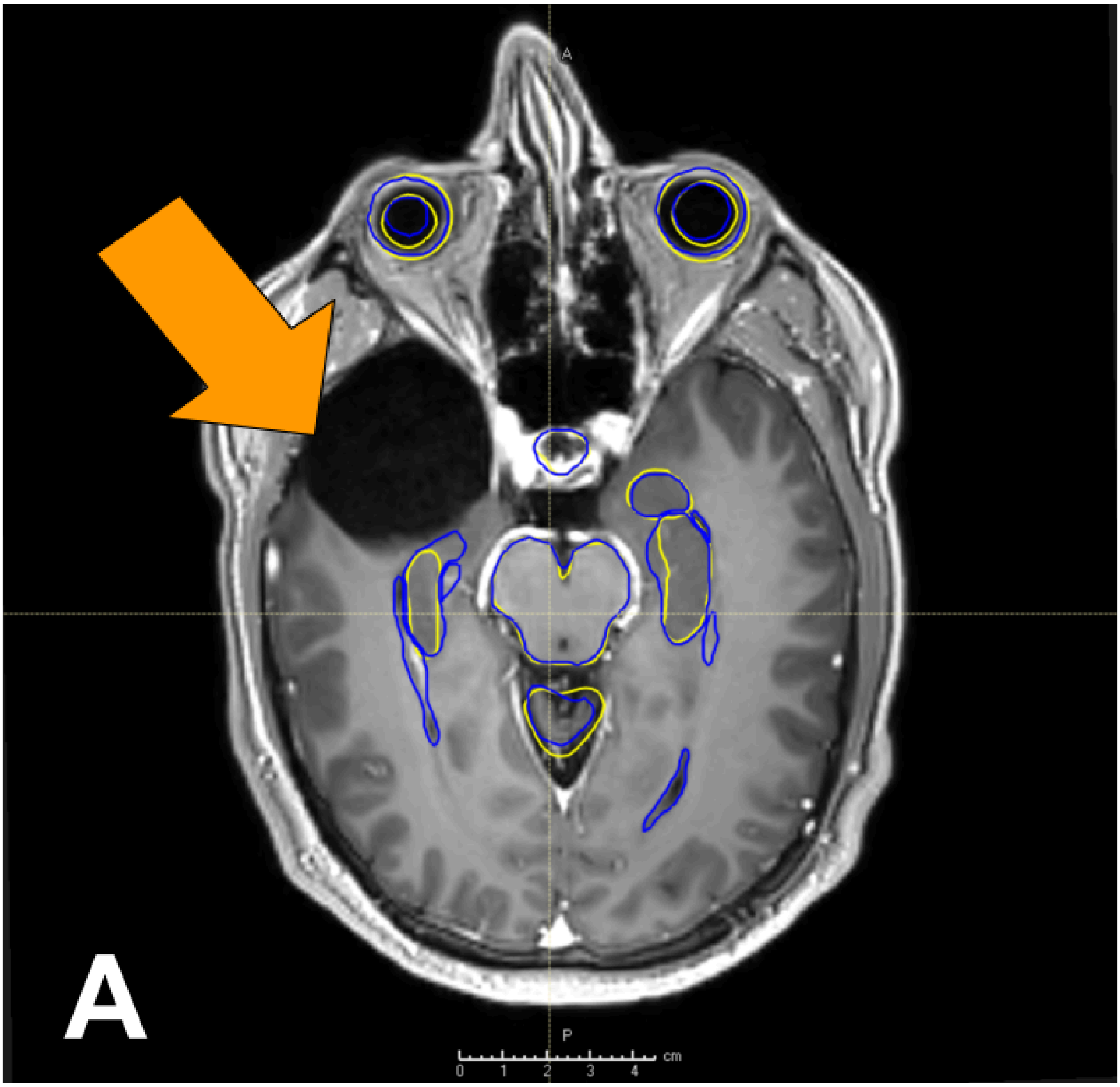

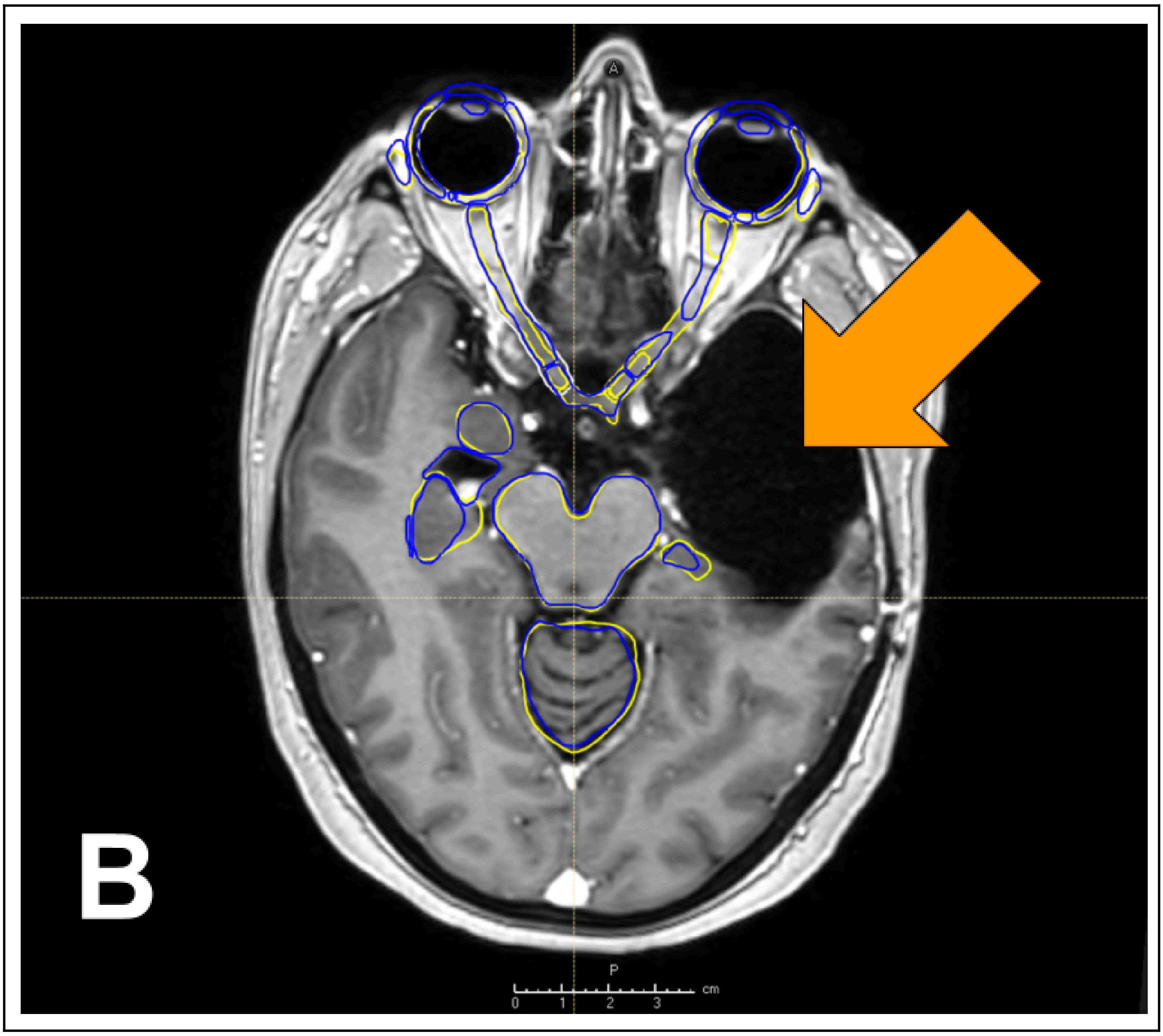
A
B
P
0 1 2 3 cm

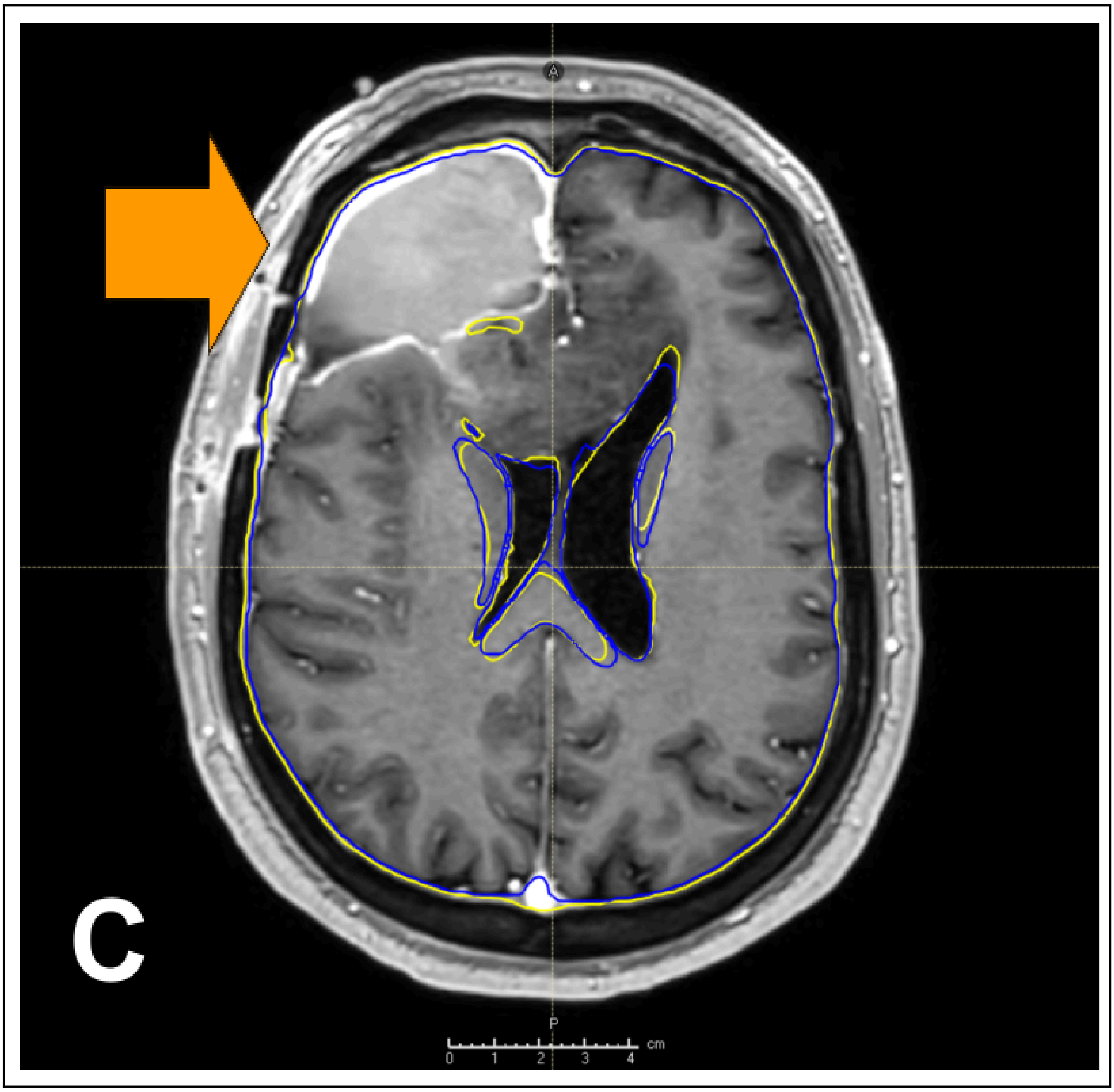

Supplementary Material A. Imaging Acquisition Parameters

Computed Tomography (CT) - CT scans were acquired on Siemens SOMATOM scanners (Confidence or Drive) with the following parameters: tube voltage of 120 kVp, slice thickness of 1 mm, in-plane pixel spacing of 0.68 mm (median; range 0.68–0.75 mm), field of view of 350 mm (median; range 348–382 mm), and soft-tissue reconstruction kernels (Qr40s or Br38s). Patient positioning was head-first supine.

T1-weighted Magnetic Resonance Imaging (MRI)- T1-weighted MRI scans were acquired on Philips scanners (Achieva, Ingenia, or Ingenia CX) using a 3D gradient echo sequence, with a slice thickness of 1 mm and in-plane pixel spacing of 1.0 mm (median; range 0.36–1.0 mm). Patient positioning was head-first supine. MRI scans were resampled to match the CT voxel spacing and image size prior to model input.

Supplementary Material B. Software specifications for metric computation (libraries, versions)

Segmentation metrics were computed using custom Python 3.10.12 scripts. The Volumetric Dice Similarity Coefficient (DSC), 95th percentile Hausdorff Distance (HD95), and Surface Dice Similarity Coefficient (SDSC) were computed using the MONAI library (v1.4.0; `DiceMetric`, `HausdorffDistanceMetric`, and `SurfaceDiceMetric` from `monai.metrics`). The Added Path Length (APL) was derived from binary boundary masks obtained via morphological erosion using SciPy (v1.13.1). Segmentation files were loaded using NiBabel (v5.2.1), and tensor operations required by MONAI were handled through PyTorch (v2.6.0). The full evaluation code, along with a Docker image for reproducibility, are made available in our GitLab repository.

Table S2. Median and interquartile range of the Dice Similarity Coefficient (DSC) for each organ and model across the 74 patients. Values are computed using reference contours after overlap removal. Bold numbers indicate the best achieved value per organ.

	T1	T1+T2	T1+T2+CT	T1+CT	Hybrid (T1+CT)
**Brain**	0.968 [0.945-0.972]	0.967 [0.944-0.973]	0.971 [0.961-0.977]	0.973 [0.964-0.976]	**0.988 [0.984-0.99]**
**Ventricles**	0.9 [0.851-0.921]	0.904 [0.849-0.92]	0.901 [0.859-0.917]	0.899 [0.865-0.916]	**0.907 [0.86-0.925]**
**Cerebellum**	0.944 [0.932-0.953]	0.947 [0.925-0.954]	0.947 [0.937-0.954]	**0.948 [0.939-0.956]**	0.944 [0.934-0.952]
**Corpus callosum**	0.878 [0.844-0.9]	0.88 [0.838-0.897]	0.88 [0.844-0.902]	0.88 [0.855-0.9]	**0.881 [0.858-0.902]**
**Orbitofrontal cortex**	0.802 [0.767-0.821]	0.798 [0.772-0.819]	0.803 [0.778-0.823]	0.806 [0.777-0.826]	**0.807 [0.773-0.828]**
**Caudate nuclei**	0.891 [0.858-0.91]	0.889 [0.858-0.908]	0.89 [0.853-0.903]	0.891 [0.853-0.905]	**0.897 [0.865-0.909]**
**Amygdalae**	0.787 [0.735-0.826]	0.787 [0.74-0.829]	0.78 [0.743-0.826]	**0.793 [0.745-0.832]**	0.787 [0.743-0.829]
**VSCCs**	0.688 [0.614-0.741]	0.7 [0.625-0.743]	0.747 [0.684-0.789]	**0.757 [0.702-0.789]**	0.746 [0.692-0.785]
**Corneas**	0.558 [0.445-0.642]	0.557 [0.457-0.629]	0.682 [0.604-0.759]	0.672 [0.616-0.751]	**0.692 [0.615-0.757]**
**Cochleas**	0.72 [0.671-0.767]	0.733 [0.675-0.78]	**0.784 [0.727-0.826]**	0.781 [0.717-0.807]	0.689 [0.0-0.755]
**Thalami**	0.888 [0.863-0.908]	0.887 [0.856-0.907]	0.889 [0.86-0.904]	**0.893 [0.87-0.905]**	**0.893 [0.86-0.904]**
**Fornices**	**0.702 [0.612-0.723]**	0.686 [0.6-0.725]	0.694 [0.609-0.724]	0.694 [0.612-0.727]	0.697 [0.611-0.731]
**Hypothalami**	0.608 [0.541-0.671]	0.608 [0.522-0.663]	**0.62 [0.562-0.683]**	0.608 [0.544-0.704]	0.603 [0.531-0.661]
**Hippocampi**	**0.829 [0.789-0.85]**	0.823 [0.781-0.85]	0.82 [0.79-0.85]	0.821 [0.791-0.848]	0.828 [0.789-0.852]
**Lacrimal glands**	0.673 [0.561-0.732]	0.674 [0.563-0.729]	0.723 [0.661-0.78]	0.729 [0.65-0.78]	**0.734 [0.667-0.788]**
**Lenses**	0.642 [0.543-0.727]	0.621 [0.544-0.7]	**0.874 [0.842-0.889]**	0.872 [0.841-0.891]	0.872 [0.843-0.889]
**Retinas**	0.711 [0.631-0.751]	0.7 [0.618-0.749]	0.792 [0.766-0.826]	0.797 [0.766-0.827]	**0.802 [0.764-0.824]**
**Maculas**	0.419 [0.293-0.536]	0.414 [0.324-0.519]	**0.648 [0.585-0.715]**	0.645 [0.579-0.718]	0.0 [0.0-0.629]
**Foveas**	0.0 [0.0-0.083]	0.0 [0.0-0.115]	0.0 [0.0-0.054]	**0.254 [0.138-0.439]**	0.0 [0.0-0.0]
**Pituitary**	0.719 [0.645-0.791]	0.717 [0.615-0.789]	**0.76 [0.681-0.829]**	**0.76 [0.661-0.823]**	0.736 [0.643-0.794]
**Pineal gland**	0.73 [0.605-0.785]	0.74 [0.649-0.809]	0.739 [0.674-0.807]	**0.747 [0.648-0.801]**	0.738 [0.617-0.808]
**Optic tracts**	0.563 [0.464-0.624]	0.574 [0.483-0.623]	0.564 [0.478-0.607]	0.555 [0.472-0.633]	**0.584 [0.478-0.636]**
**Optic nerves**	0.721 [0.642-0.759]	0.71 [0.633-0.754]	0.83 [0.811-0.848]	**0.833 [0.815-0.847]**	0.711 [0.645-0.748]
**Brainstem**	0.926 [0.912-0.934]	0.926 [0.908-0.933]	**0.927 [0.907-0.936]**	0.926 [0.911-0.937]	0.924 [0.911-0.936]
**Optic chiasm**	0.739 [0.678-0.783]	0.734 [0.687-0.777]	0.747 [0.686-0.779]	0.738 [0.692-0.78]	**0.752 [0.661-0.79]**

Table S3. Median and interquartile range of the surface Dice Similarity Coefficient (sDSC) for each organ and model across the 74 patients. Values are computed using reference contours after overlap removal. Bold numbers indicate the best achieved value per organ.

	T1	T1+T2	T1+T2+CT	T1+CT	Hybrid (T1+CT)
**Brain**	0.803 [0.761-0.834]	0.8 [0.753-0.84]	0.843 [0.793-0.865]	0.843 [0.789-0.866]	**0.894 [0.849-0.933]**
**Ventricles**	0.924 [0.878-0.945]	0.927 [0.866-0.949]	0.924 [0.856-0.942]	0.924 [0.872-0.944]	**0.929 [0.877-0.95]**
**Cerebellum**	0.71 [0.643-0.765]	0.721 [0.606-0.773]	**0.738 [0.65-0.772]**	0.735 [0.666-0.778]	0.725 [0.642-0.754]
**Corpus callosum**	0.882 [0.832-0.911]	0.874 [0.801-0.914]	**0.883 [0.823-0.913]**	0.875 [0.838-0.919]	**0.883 [0.839-0.917]**
**Orbitofrontal cortex**	0.793 [0.754-0.831]	0.797 [0.756-0.826]	0.799 [0.762-0.831]	0.799 [0.764-0.831]	**0.81 [0.774-0.842]**
**Caudate nuclei**	**0.914 [0.86-0.943]**	0.905 [0.847-0.937]	0.903 [0.845-0.935]	0.906 [0.847-0.94]	0.913 [0.86-0.947]
**Amygdalae**	0.747 [0.655-0.821]	0.74 [0.655-0.83]	0.735 [0.65-0.812]	0.747 [0.65-0.817]	**0.752 [0.684-0.818]**
**VSCCs**	0.675 [0.596-0.737]	0.683 [0.583-0.744]	0.78 [0.703-0.824]	**0.784 [0.685-0.84]**	0.748 [0.664-0.801]
**Corneas**	0.612 [0.503-0.714]	0.623 [0.505-0.7]	**0.775 [0.681-0.869]**	0.769 [0.675-0.841]	0.77 [0.693-0.851]
**Cochleas**	0.824 [0.723-0.875]	0.841 [0.742-0.899]	**0.911 [0.835-0.945]**	0.897 [0.826-0.942]	0.75 [0.0-0.885]
**Thalami**	0.757 [0.676-0.826]	0.756 [0.676-0.81]	0.76 [0.668-0.795]	**0.767 [0.697-0.821]**	0.762 [0.675-0.808]
**Fornices**	0.888 [0.815-0.914]	0.885 [0.799-0.918]	0.885 [0.808-0.917]	0.881 [0.822-0.91]	**0.889 [0.828-0.919]**
**Hypothalami**	0.689 [0.607-0.752]	0.676 [0.599-0.78]	**0.709 [0.612-0.785]**	0.707 [0.616-0.799]	0.7 [0.589-0.764]
**Hippocampi**	0.854 [0.796-0.887]	0.846 [0.795-0.882]	**0.859 [0.786-0.885]**	0.848 [0.796-0.891]	**0.859 [0.792-0.89]**
**Lacrimal glands**	0.656 [0.554-0.743]	0.652 [0.531-0.724]	0.721 [0.651-0.834]	0.749 [0.644-0.82]	**0.755 [0.658-0.812]**
**Lenses**	0.646 [0.492-0.786]	0.634 [0.532-0.755]	**0.981 [0.941-0.988]**	0.98 [0.948-0.989]	0.975 [0.947-0.988]
**Retinas**	0.84 [0.757-0.882]	0.832 [0.759-0.874]	0.929 [0.9-0.95]	**0.931 [0.897-0.946]**	0.921 [0.89-0.945]
**Maculas**	0.694 [0.523-0.821]	0.693 [0.563-0.823]	0.89 [0.848-0.957]	**0.91 [0.853-0.955]**	0.0 [0.0-0.889]
**Foveas**	0.2 [0.0-0.446]	0.186 [0.0-0.496]	0.0 [0.0-0.494]	**0.667 [0.533-0.815]**	0.0 [0.0-0.0]
**Pituitary**	0.744 [0.617-0.872]	0.721 [0.611-0.866]	**0.804 [0.637-0.911]**	0.803 [0.624-0.889]	0.77 [0.615-0.862]
**Pineal gland**	0.856 [0.686-0.944]	0.864 [0.725-0.957]	**0.897 [0.803-0.948]**	0.868 [0.779-0.93]	0.884 [0.679-0.955]
**Optic tracts**	**0.777 [0.645-0.829]**	0.763 [0.635-0.818]	0.766 [0.672-0.81]	0.763 [0.658-0.832]	0.765 [0.646-0.843]
**Optic nerves**	0.806 [0.715-0.865]	0.795 [0.708-0.862]	**0.944 [0.914-0.955]**	**0.944 [0.923-0.959]**	0.79 [0.725-0.853]
**Brainstem**	0.772 [0.693-0.819]	0.767 [0.671-0.818]	0.786 [0.68-0.824]	**0.781 [0.67-0.83]**	0.77 [0.689-0.828]
**Optic chiasm**	0.903 [0.858-0.942]	0.897 [0.854-0.941]	0.905 [0.856-0.949]	0.904 [0.849-0.941]	**0.908 [0.854-0.945]**

Table S4. Median and interquartile range of the Added Path Length (APL) in mm, for each organ and model across the 74 patients. Values are computed using reference contours after overlap removal. Bold numbers indicate the best achieved value per organ.

	T1	T1+T2	T1+T2+CT	T1+CT	Hybrid (T1+CT)
**Brain**	65840 [58230-75546]	66117 [59507-75420]	58325 [50182-68302]	57072 [49293-68698]	**36650 [33162-42094]**
**Ventricles**	6116 [5015-7911]	6134 [5150-7662]	6157 [5172-7894]	5916 [4993-7557]	**5906 [4957-7085]**
**Cerebellum**	12737 [11400-14024]	12494 [11164-13898]	**12009 [11079-13549]**	12179 [11207-13557]	12751 [11277-13594]
**Corpus callosum**	4475 [3773-4906]	4510 [3824-5038]	4567 [3932-5207]	4441 [3833-4944]	**4345 [3751-5004]**
**Orbitofrontal cortex**	7438 [6809-8488]	7575 [6690-8664]	**7311 [6535-8118]**	7427 [6570-8231]	7427 [6766-8559]
**Caudate nuclei**	1502 [1326-1653]	1523 [1325-1730]	1552 [1297-1733]	1547 [1341-1709]	**1453 [1285-1676]**
**Amygdalae**	730 [622-845]	**717 [629-855]**	730 [639-849]	728 [626-843]	719 [614-821]
**VSCCs**	591 [499-703]	582 [507-670]	539 [465-618]	**523 [470-623]**	554 [508-618]
**Corneas**	751 [629-853]	724 [632-839]	613 [520-805]	**611 [511-779]**	656 [551-775]
**Cochleas**	187 [165-230]	187 [152-220]	160 [140-195]	170 [145-199]	**137 [0-182]**
**Thalami**	**2474 [2250-2870]**	2565 [2258-2795]	2578 [2309-2925]	2505 [2242-2785]	2505 [2262-2910]
**Fornices**	612 [530-677]	598 [517-691]	**591 [525-680]**	603 [531-673]	593 [518-707]
**Hypothalami**	561 [439-634]	**539 [432-642]**	571 [480-638]	572 [462-641]	570 [475-637]
**Hippocampi**	1818 [1479-2121]	1834 [1531-2094]	1734 [1577-2034]	**1728 [1530-2089]**	1826 [1570-2198]
**Lacrimal glands**	624 [523-737]	623 [534-739]	**578 [476-696]**	600 [492-686]	581 [530-731]
**Lenses**	266 [237-310]	270 [225-297]	**134 [117-164]**	137 [116-162]	135 [116-162]
**Retinas**	4456 [4004-5113]	4466 [4096-4983]	**3680 [3289-4270]**	3766 [3324-4279]	3685 [3413-4411]
**Maculas**	113 [94-129]	108 [93-126]	73 [58-85]	77 [60-87]	**0 [0-71]**
**Foveas**	9 [6-10]	8 [5-9]	0 [0-5]	6 [4-9]	**0 [0-0]**
**Pituitary**	260 [219-314]	260 [214-317]	255 [199-313]	**242 [195-314]**	248 [213-315]
**Pineal gland**	114 [84-137]	115 [81-132]	**98 [73-134]**	108 [82-127]	118 [83-140]
**Optic tracts**	**231 [200-285]**	243 [203-282]	242 [195-300]	241 [203-279]	237 [204-283]
**Optic nerves**	920 [806-1070]	930 [813-1074]	610 [541-687]	**603 [534-665]**	940 [818-1058]
**Brainstem**	3823 [3501-4273]	3855 [3497-4275]	3820 [3450-4177]	3800 [3472-4182]	**3788 [3456-4290]**
**Optic chiasm**	241 [192-309]	239 [183-306]	236 [183-287]	**233 [179-300]**	237 [195-296]

Table S5. Median and interquartile range [IQR] of the Hausdorff Distance at percentile 95% (HD95) for each organ and model across the 74 patients. Values are computed using reference contours after overlap removal. Bold numbers indicate the best achieved value per organ.

	T1	T1+T2	T1+T2+CT	T1+CT	Hybrid (T1+CT)
**Brain**	4.123 [3.201-5.314]	4.062 [3.162-5.196]	3.606 [3.0-4.663]	3.464 [3.0-4.472]	**2.639 [2.0-3.606]**
**Ventricles**	**1.732 [1.414-3.742]**	**1.732 [1.414-3.0]**	2.0 [1.414-4.123]	2.0 [1.414-3.464]	2.0 [1.414-3.0]
**Cerebellum**	3.317 [3.0-4.583]	3.317 [2.957-4.265]	3.317 [2.828-4.243]	**3.162 [3.0-3.606]**	3.464 [3.0-4.243]
**Corpus callosum**	2.449 [2.0-3.278]	2.639 [2.0-3.606]	**2.236 [2.0-3.278]**	**2.236 [2.0-3.0]**	**2.236 [2.0-3.0]**
**Orbitofrontal cortex**	3.0 [2.289-4.213]	3.0 [2.449-4.527]	3.0 [2.449-4.472]	3.0 [2.449-4.555]	**2.828 [2.236-4.123]**
**Caudate nuclei**	1.732 [1.414-2.449]	1.732 [1.414-2.648]	1.866 [1.414-2.236]	1.846 [1.414-2.236]	**1.414 [1.414-2.236]**
**Amygdalae**	2.449 [2.0-3.57]	2.449 [2.0-3.162]	2.449 [2.0-3.116]	2.449 [2.0-3.427]	**2.444 [2.0-3.317]**
**VSCCs**	2.828 [2.236-3.278]	2.639 [2.236-3.162]	**2.236 [2.0-2.449]**	**2.236 [2.0-2.828]**	**2.236 [2.236-2.828]**
**Corneas**	4.243 [3.464-5.172]	4.123 [3.317-5.477]	3.0 [2.236-4.123]	3.0 [2.236-4.092]	**2.828 [2.236-3.742]**
**Cochleas**	2.0 [1.732-2.449]	2.0 [1.414-2.449]	**1.414 [1.414-2.0]**	1.613 [1.414-2.0]	1.732 [1.414-2.236]
**Thalami**	2.828 [2.236-3.742]	2.639 [2.236-3.742]	**2.449 [2.236-3.57]**	**2.449 [2.236-3.162]**	2.639 [2.059-3.606]
**Fornices**	2.236 [1.7-2.828]	2.236 [1.732-3.0]	2.236 [1.732-3.0]	2.236 [1.732-3.0]	**2.0 [1.732-2.991]**
**Hypothalami**	4.062 [2.629-8.483]	4.243 [3.0-9.154]	4.123 [2.449-8.136]	**4.0 [2.236-7.778]**	**4.0 [2.449-8.061]**
**Hippocampi**	2.449 [2.0-3.742]	2.639 [2.236-3.708]	2.639 [2.059-3.57]	2.639 [2.0-3.742]	**2.343 [2.0-3.708]**
**Lacrimal glands**	4.292 [3.0-6.403]	4.472 [3.0-6.164]	3.871 [2.236-5.787]	3.674 [2.544-6.0]	**3.447 [2.236-5.333]**
**Lenses**	2.724 [2.0-3.708]	2.914 [2.236-3.742]	**1.0 [1.0-1.414]**	**1.0 [1.0-1.414]**	**1.0 [1.0-1.414]**
**Retinas**	2.0 [1.732-2.396]	2.236 [1.732-2.828]	**1.414 [1.414-1.732]**	**1.414 [1.414-1.653]**	**1.414 [1.414-1.732]**
**Maculas**	2.449 [2.124-3.297]	2.449 [2.236-3.162]	**1.414 [1.414-2.0]**	**1.414 [1.414-2.0]**	**1.414 [1.311-1.749]**
**Foveas**	3.093 [2.3-4.043]	3.127 [2.449-4.015]	2.177 [1.649-2.605]	2.071 [1.529-2.42]	**2.0 [1.414-2.3]**
**Pituitary**	2.449 [2.0-4.0]	2.449 [2.0-3.806]	**2.236 [1.732-3.606]**	**2.236 [1.732-3.742]**	2.828 [2.0-3.598]
**Pineal gland**	2.0 [1.414-3.162]	2.0 [1.414-3.0]	**1.866 [1.414-2.236]**	2.0 [1.414-2.828]	1.96 [1.414-3.606]
**Optic tracts**	4.917 [2.828-6.956]	4.826 [2.828-6.481]	4.472 [3.162-6.403]	**4.359 [2.449-6.403]**	4.472 [2.449-6.325]
**Optic nerves**	2.236 [2.0-3.0]	2.305 [2.0-3.079]	**1.414 [1.414-2.0]**	**1.414 [1.414-2.0]**	2.449 [2.0-3.0]
**Brainstem**	**3.0 [2.289-3.742]**	**3.0 [2.236-3.78]**	**3.0 [2.236-3.901]**	**3.0 [2.236-4.0]**	**3.0 [2.449-4.0]**
**Optic chiasm**	2.112 [1.414-3.162]	2.236 [1.414-3.427]	2.236 [1.414-3.579]	2.236 [1.732-3.185]	**2.0 [1.414-3.13]**

Table S6. Median of the DSC and sDSC for the T1+CT model calculated using the reference contours without and with overlap (w. overlap), as well as the difference in the medians.

	DSC	DSC (w. overlap)	Difference in median	*sDSC*	*sDSC* (w. overlap)	Difference in median
**Brain**	0.973	0.896	0.077	0.843	0.67	0.173
**Ventricles**	0.899	0.888	0.011	0.924	0.898	0.026
**Cerebellum**	0.948	0.947	0.001	0.735	0.732	0.003
**Corpus callosum**	0.88	0.88	0	0.875	0.874	0.001
**Orbitofrontal cortex**	0.806	0.806	0	0.799	0.799	0
**Caudate nuclei**	0.891	0.891	0	0.906	0.906	0
**Amygdalae**	0.793	0.797	-0.004	0.747	0.752	-0.005
**VSCCs**	0.757	0.755	0.002	0.784	0.781	0.003
**Corneas**	0.672	0.669	0.003	0.769	0.751	0.018
**Cochleas**	0.781	0.781	0	0.897	0.897	0
**Thalami**	0.893	0.893	0	0.767	0.759	0.008
**Fornices**	0.694	0.683	0.011	0.881	0.863	0.018
**Hypothalami**	0.608	0.61	-0.002	0.707	0.698	0.009
**Hippocampi**	0.821	0.821	0	0.848	0.848	0
**Lacrimal glands**	0.729	0.732	-0.003	0.749	0.723	0.026
**Lenses**	0.872	0.872	0	0.98	0.98	0
**Retinas**	0.797	0.793	0.004	0.931	0.926	0.005
**Maculas**	0.645	0.672	-0.027	0.91	0.906	0.004
**Foveas**	0.254	0.254	0	0.667	0.667	0
**Pituitary**	0.76	0.76	0	0.803	0.803	0
**Pineal gland**	0.747	0.75	-0.003	0.868	0.871	-0.003
**Optic tracts**	0.555	0.565	-0.01	0.763	0.756	0.007
**Optic nerves**	0.833	0.833	0	0.944	0.943	0.001
**Brainstem**	0.926	0.926	0	0.781	0.781	0
**Optic chiasm**	0.738	0.738	0	0.904	0.904	0

Table S7. Number of patients (N) with non-zero overlap removal and median percentage and interquartile range [IQR] of volume removed by overlap removal in the reference contours, per organ, across 74 patients.

organ	N	Median [IQR] (%)
**Brain**	74	16.66 [15.45-17.51]
**Ventricles**	69	7.51 [4.34-10.19]
**Cerebellum**	62	0.19 [0.04-0.28]
**Corpus callosum**	30	0.0 [0.0-0.04]
**Orbitofrontal cortex**	14	0.0 [0.0-0.0]
**Caudate nuclei**	6	0.0 [0.0-0.0]
**Amygdalae**	52	0.53 [0.0-2.38]
**VSCCs**	56	0.45 [0.03-1.02]
**Corneas**	74	8.02 [6.08-9.49]
**Cochleas**	1	0.0 [0.0-0.0]
**Thalami**	74	0.57 [0.28-1.51]
**Fornices**	73	7.19 [4.31-10.32]
**Hypothalami**	69	0.89 [0.33-1.82]
**Hippocampi**	3	0.0 [0.0-0.0]
**Lacrimal glands**	70	2.95 [1.23-5.51]
**Lenses**	3	0.0 [0.0-0.0]
**Retinas**	74	2.17 [1.9-2.35]
**Maculas**	70	6.0 [5.32-6.92]
**Foveas**	0	0.0 [0.0-0.0]
**Pituitary**	1	0.0 [0.0-0.0]
**Pineal gland**	32	0.0 [0.0-1.63]
**Optic tracts**	60	0.83 [0.14-2.1]
**Optic nerves**	67	0.3 [0.14-0.47]
**Brainstem**	0	0.0 [0.0-0.0]
**Optic chiasm**	0	0.0 [0.0-0.0]

Table S8. Mean and standard deviation (Std) of the reference contours volume (after overlap removal), expressed in number of voxels, for each of the 25 organs across all 74 patients. Structures are ordered from smallest to largest.

Structure	Mean (#voxels)	Std
**Foveas**	14	4
**Maculas**	241	59
**Pineal Gland**	471	208
**Optic Tracts**	738	270
**Cochleas**	797	251
**Lenses**	940	306
**Optic Chiasm**	968	255
**Pituitary Gland**	1,324	789
**Hypothalami**	1,923	675
**Fornices**	2,062	457
**Corneas**	2,530	749
**Vsccs**	2,628	922
**Lacrimal Glands**	3,031	1,124
**Optic Nerves**	3,731	932
**Amygdalae**	3,934	1,104
**Hippocampi**	11,174	2,439
**Caudate Nuclei**	12,946	2,637
**Retinas**	16,540	1,496
**Thalami**	26,419	3,979
**Corpus Callosum**	36,849	8,806
**Orbitofrontal Cortex**	45,424	10,533
**Brainstem**	58,368	8,642
**Ventricles**	74,836	45,011
**Cerebellum**	256,876	100,715
**Brain**	2,511,898	283,607

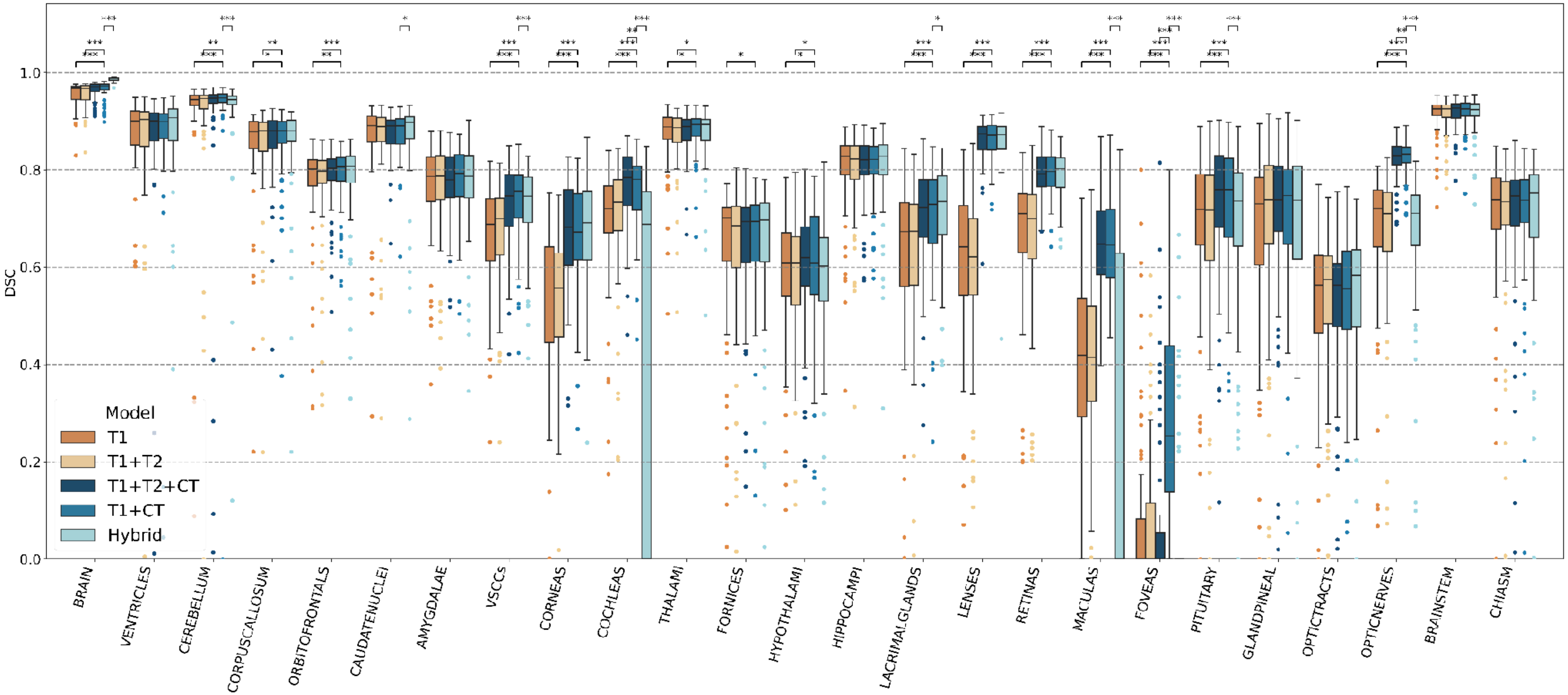

Figure S2. Boxplots of Dice similarity coefficient (DSC) per organ for all evaluated models. Organs are displayed from low to high priority, according to the structure-priority scheme defined by the clinical experts to minimize information loss in overlapping regions. Note that the DSC were computed using reference contours after overlap removal. Each box represents the distribution across patients, with the median shown as a horizontal line, boxes indicating the interquartile range, and whiskers extending to 1.5× the interquartile range; outliers are shown as individual points. Statistical significance of pairwise comparisons between T1 + CT model (chosen as reference for comparison) and the other models is indicated by horizontal brackets and asterisks (* $p < 0.05$, ** $p < 0.01$, *** $p < 0.001$), based on paired Wilcoxon signed-rank tests

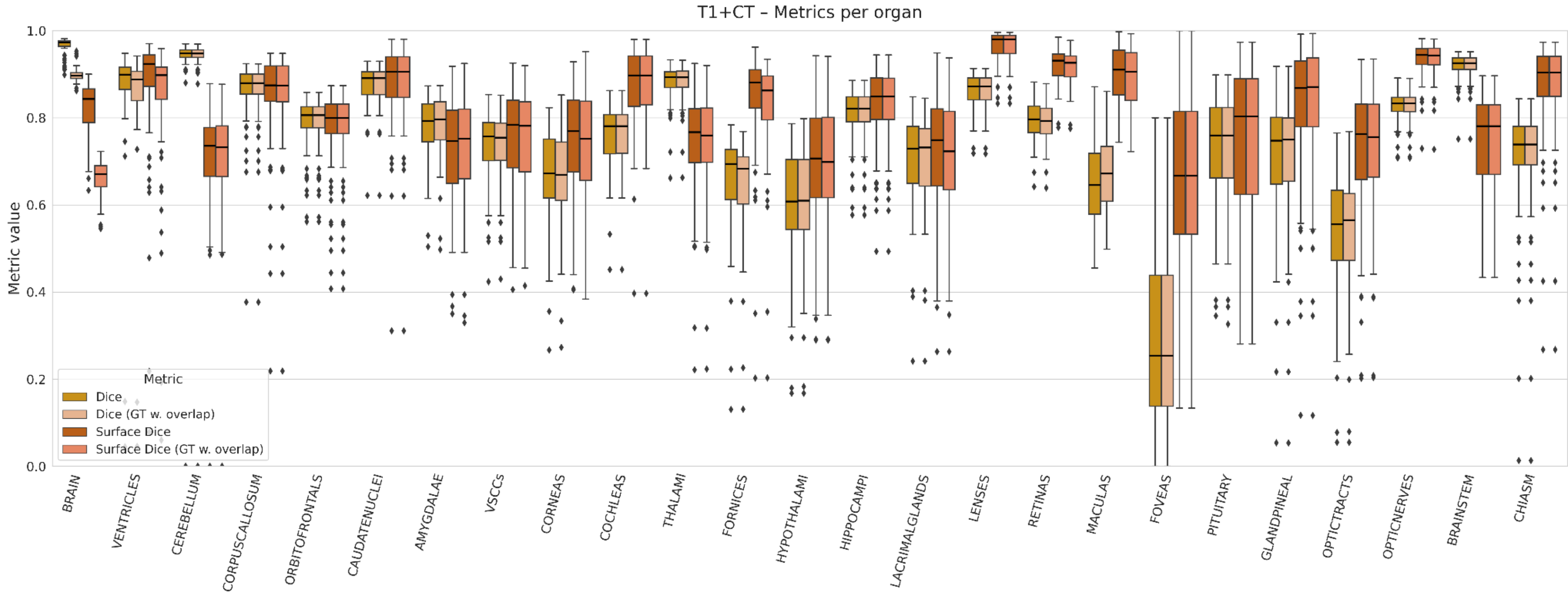

Figure S3. Boxplots of DSC and surface DSC similarity metrics per organ for the T1 + CT model, computed using reference contours with structure overlap (GT w. overlap) and without. The median is indicated by a horizontal line, boxes represent the interquartile range, and whiskers extend to 1.5 × the interquartile range; outliers are shown as individual points. Organs are displayed in the same order used for nnU-Net training, reflecting the structure-priority scheme defined by clinical experts to minimize information loss in regions where contours may overlap

Supplementary Material C. Training stability analysis of the CT Hybrid model

The CT Hybrid model was designed to leverage CT specifically for the segmentation of CT-visible structures (brain, VSCCs, corneas, cochleas, lacrimal glands, lenses, retinas, maculas, and foveas). However, it showed unexpectedly poor performance for cochleas (median DSC = 0.689, IQR 0.0–0.755), maculas (median DSC = 0.000, IQR 0.0–0.629), and foveas (median DSC = 0.000, IQR 0.0–0.0), despite the T1+CT model achieving 0.781, 0.645, and 0.254 respectively for these same structures. The wide IQR and zero medians reflect high variability across folds as detailed in Table S2. Per-fold analysis of the training logs revealed that this underperformance might be attributable to training instability caused by extreme class imbalance (Table S9, Figures S4, S5).

Table S9. Mean DSC per cross-validation fold for cochleas, maculas, and foveas in the CT Hybrid model.

	COCHLEAS	MACULAS	FOVEAS
**Fold 0**	0.000	0.566	0.297
**Fold 1**	0.758	0.690	0.000
**Fold 2**	0.000	0.000	0.000
**Fold 3**	0.735	0.000	0.000
**Fold 4**	0.727	0.000	0.000

Figure S5 shows distinct failure patterns for the CT Hybrid model during training across structures and folds, in contrast to the T1+CT model, which shows a stable behaviour (Figure S4). In the CT Hybrid model, cochleas are learned during early training (pseudo-Dice[1] ~0.7) but undergoes catastrophic forgetting by the end of training in folds 0 and 2, where the final DSC are both 0.000. For maculas, a similar pattern of early learning followed by complete forgetting is observed in folds 2, 3 and 4. For foveas, the failure mode is different: foveas are never stably learned in folds 2, 3, and 4 (the pseudo-Dice remains at zero throughout training) and only transiently predicted in fold 1. This might be explained by the extremely small volume of the fovea (~14 voxels on average, Table S8), which might cause this training instability / failure with respect to the T1+CT model. Actually, there is an extreme volume imbalance between structures in the CT Hybrid model: the Brain structure (~3M voxels) accounts for >99% of all foreground voxels, with no intermediate-sized structures to bridge the gap to the smallest ones. As the learning rate decays, the gradient might be almost entirely dominated by the brain, causing the model to abandon small-structure segmentation. In contrast, the T1+CT model includes 25 structures spanning a broad range of sizes (Table S8), providing a more balanced gradient signal throughout training. As shown in Figure S4, all three structures are stably retained across all 5 folds in the T1+CT model, with no catastrophic forgetting observed.

[1] The pseudo-Dice is an approximation of the Dice similarity coefficient computed by nnU-Net on randomly sampled training patches during each epoch, rather than on full images. It serves as a training progress indicator and differs from the final evaluation DSC, which is computed on complete images on unseen data.

Figure S4. Per-epoch pseudo-Dice during training for cochleas, maculas, and foveas across all five cross-validation folds of the T1+CT model (extracted from the training logs from nnU-Net). Solid lines represent the raw per-epoch pseudo-Dice computed on a single training batch; dashed lines indicate the final mean DSC obtained at evaluation on unseen data.

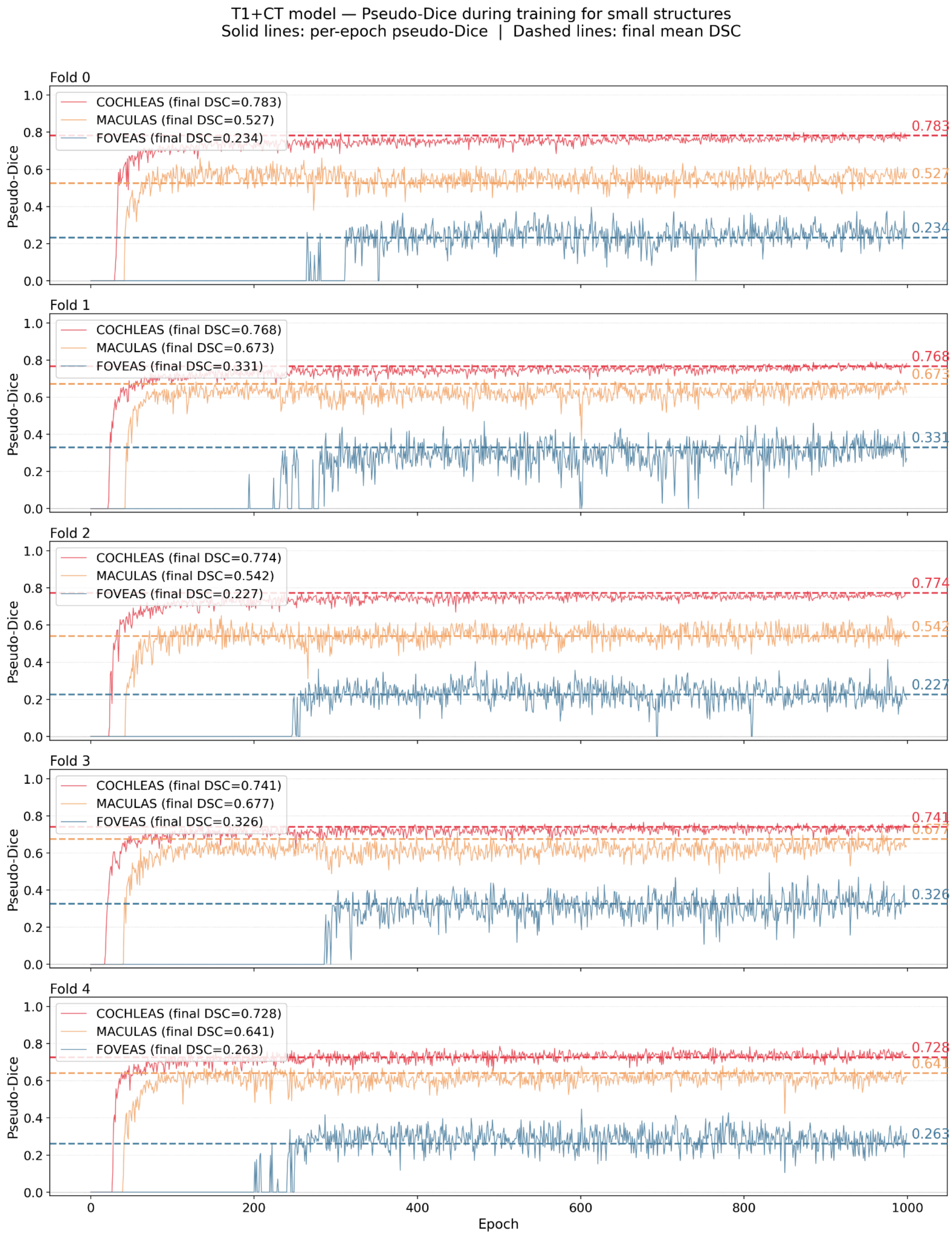

Figure S5. Per-epoch pseudo-Dice during training for cochleas, maculas, and foveas across all five cross-validation folds of the CT-only counterpart of the Hybrid model (extracted from the training logs from nnU-Net). Solid lines represent the raw per-epoch pseudo-Dice computed on a single training batch; dashed lines indicate the final mean DSC obtained at evaluation on unseen data.

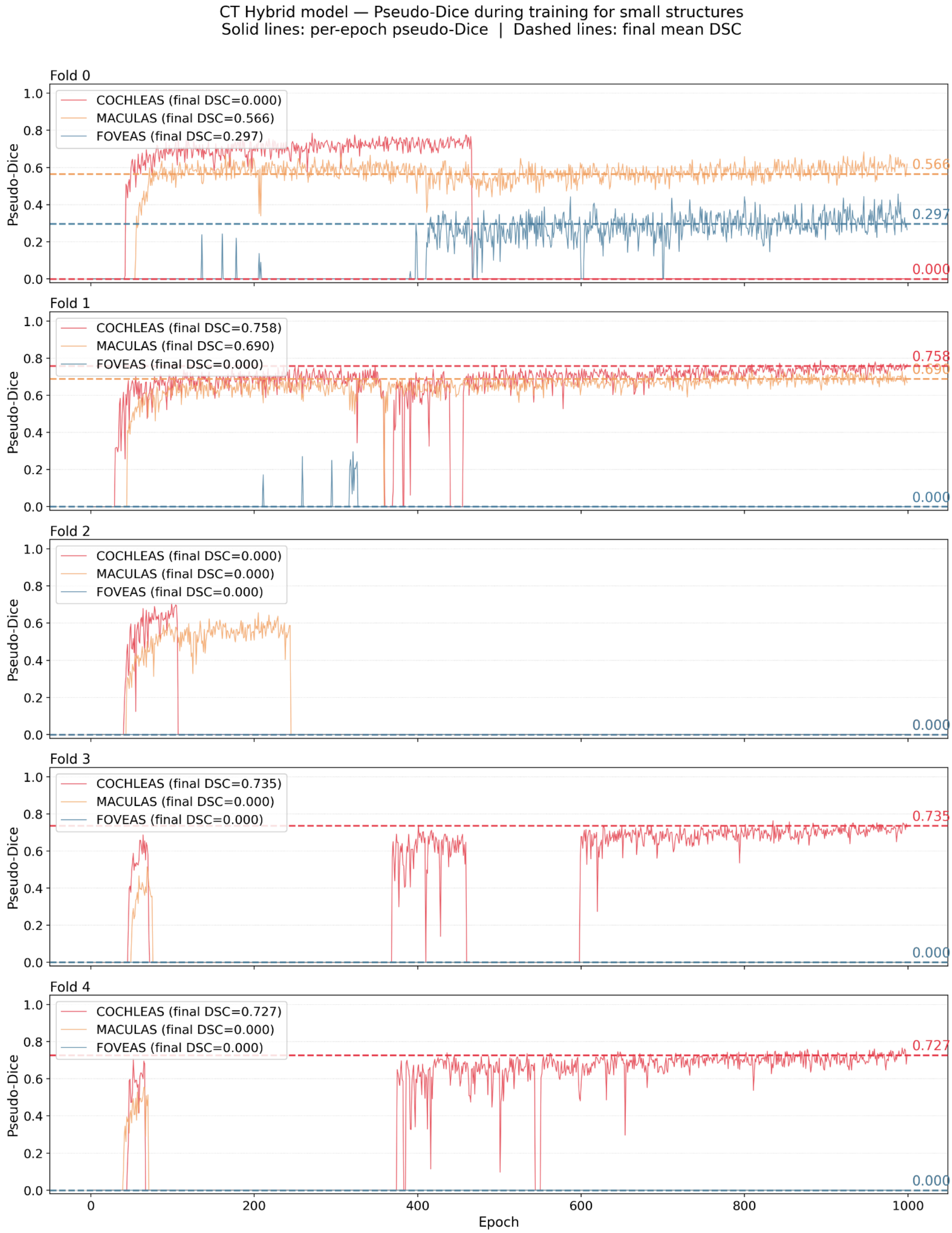

Supplement: Supplementary file 1 [file mmc1.pdf]
